# Mindfulness and Acceptance as Potential Protective Factors for Mothers of Children With Fragile X Syndrome

**DOI:** 10.3389/fpubh.2018.00316

**Published:** 2018-11-06

**Authors:** Anne C. Wheeler, Shari Miller, Amanda Wylie, Anne Edwards

**Affiliations:** RTI International, Durham, NC, United States

**Keywords:** *FMR1* premutation, mindfulness, mindful parenting, acceptance, fragile X syndrome

## Abstract

Women with an *FMR1* premutation may be at increased genetic risk for stress vulnerability. This increased vulnerability, when combined with stressful parenting that can result from raising children with fragile X syndrome (FXS), may result in negative physical and emotional outcomes. Mindfulness and acceptance have been found to be protective factors for parents of children with similar behavioral challenges, but these traits have not previously been explored among mothers with a child with FXS. This study explored the associations of child disability severity with maternal stress, anxiety, depression, and physical health symptoms in 155 biological mothers of children with FXS. Women completed an online survey using standardized measures of stress, mindfulness, and acceptance. General mindfulness, mindfulness in the parenting role, and general acceptance were explored as potential protective factors between the child disability severity and maternal outcomes. Trait mindfulness and acceptance were significant predictors of lower stress, anxiety, depression, and daily health symptoms, while mindful parenting was associated with lower stress, anxiety, and depression. Acceptance was found to attenuate the effects of child severity on maternal stress and depression. These findings suggest that interventions focused on improving mindfulness and acceptance may promote health and well-being for mothers of children with FXS and have important health implications for all individuals with an *FMR1* premutation.

## Introduction

Parenting a child with a developmental disability, especially one with associated challenging behaviors and long-term needs, can have a negative impact on health and well-being for family members (1). There is some emerging evidence that this may be especially true for biological mothers of children with fragile X syndrome (FXS). FXS, which results from a trinucleotide repeat expansion (CGG) of over 200 repeats at Xq27.3 on the upper end of the *FMR1* gene, is the most common inherited form of intellectual disability and is highly co-morbid with autism, anxiety, aggression, and ADHD (2). As many as 30% of males with FXS engage in aggressive behaviors severe enough to cause injury to a parent or sibling (3, 4), adding to significant parental stress. Although both parents are affected by stress related to caregiving, biological mothers of children with FXS may be especially vulnerable. Biological mothers of children with FXS are almost always carriers of an *FMR1* expansion, usually in the premutation form (defined as having 55–200 CGG repeats). It is now accepted that individuals with a premutation are at increased risk for several health problems. Two known conditions—fragile X–associated premature ovarian insufficiency and fragile X–associated tremor ataxia syndrome—are present in around a third of individuals with an *FMR1* premutation (5, 6), and increased rates of a host of other physical, emotional, cognitive, and reproductive challenges have been reported (7). This may be especially true for women with mid-range (~80–100) premutation expansions (8–14). Recent studies also suggest that premutation carriers are at heightened risk for stress-related illnesses (15, 16) as the result of the disruption of *FMR1* protein development and possible subsequent differences in HPA axis regulation (15, 17). This increased stress vulnerability may lead to more adverse mental and physical health outcomes for mothers (8–10, 18, 19), in response to behavior problems in their children (20). This dual vulnerability of known genetic risk coupled with the chronic elevated stress related to raising a child with significant behavioral and developmental challenges can negatively impact well-being for both parent and child and may increase risks for later-onset fragile X–associated conditions.

Because of this growing concern, researchers and clinicians are publicly calling for innovative, empirically based stress reduction interventions for this population (21). Third-wave therapies, such as acceptance and commitment therapy [ACT; (22)], and mindfulness-based therapies (23, 24) hold promise as methods to reduce stress in caregivers of individuals with intellectual or developmental disabilities (25–30). A convincing body of evidence shows that mindfulness interventions are effective in reducing depression, anxiety (31), substance abuse (32), stress (33, 34), and insomnia (35). Most relevant for parents managing a potentially difficult diagnosis in their child, mindfulness and acceptance intervention techniques can promote psychological flexibility and acceptance (36), increase parent satisfaction with their parenting skills (29), and decrease challenging behavior in the children (29, 37).

These findings have intrigued researchers and clinicians who support parents of children with disabilities (26) and has led to increases in research to understand the potential protective role of acceptance and mindfulness for these parents. Recent studies explored the mediating role of acceptance and mindfulness processes in parents of children with autism spectrum disorder, intellectual disability (38–41), or both. Findings from these studies showed that both acceptance and mindfulness significantly mediated the relationship between child behavior and parental well-being. Should these processes hold true for mothers of children with FXS, it could provide promising directions for intervention development for this population.

The goal of the current study was to extend previous research on mediating processes (acceptance, trait mindfulness, and mindful parenting) on the relationship between the severity of child disability and maternal outcomes to mothers of children with FXS. We hypothesized that the degree of child disability severity would predict parents' use of mindfulness techniques, and that varying mindfulness techniques would serve to attenuate the effect of the severity of children's disability on maternal outcomes including stress, anxiety, depression, and health symptoms. By identifying psychological processes such as acceptance and mindfulness, which may serve as protective factors for these women, we can better design interventions for reducing stress and protecting the health of mothers with an *FMR1* premutation.

## Materials and methods

### Design

A national survey registry was used to recruit and administer published measures of stress, anxiety, depression, physical health symptoms, trait mindfulness, mindful parenting, and acceptance to biological mothers of at least one child with FXS. This study is part of a larger survey project exploring stress, coping, and mindfulness among women with an *FMR1* premutation.

### Participants

Following Institutional Review Board approval, women were recruited from the *Our Fragile X World* (https://www.ourfragilexworld.org) registry, a national registry focused specifically on experiences of individuals with FXS and their families. A total of 166 women completed this survey; 11 mothers reported having the full mutation and were removed from subsequent analysis. The respondents were primarily white (90%) and well-educated (67% with at least a 4-year college degree), with an average age of 49.7 years (SD = 7.0). Family income was also generally high, with nearly 40% reporting annual incomes over $100,000. Most mothers (82%) in this sample had a premutation, though 11% were not tested. Demographic information for participants is reported in Table 1.

**Table 1:** Demographics of the sample.

	***N***	**Mean (SD)**
Mother's age	153	49.7 (7.0)
Total number of children in family	155	2.1 (0.9)
Total number of children with FM or PM in the family	155	1.4 (0.6)
Total number of children with FM in the family	155	1.3 (0.6)
Number of co-occurring conditions in family	155	6.4 (3.7)
Child's age	321	18.5 (6.4)
Number of co-occurring conditions among children	322	3.1 (2.6)
	***N***	**%**
Mother's race (*n* = 155)	
White	136	90.1
Child's gender (*n* = 322)	
Female	113	35.1
Mother's Education (*n* = 155)	
High school or less	11	7.1
Some college	22	14.2
2-year degree	20	12.9
4-year degree	57	36.8
Graduate or prof degree	45	29.0
Family income (*n* = 155)	
< $25,000	11	7.1
$25,000–50,000	25	16.1
$50,001–75,000	27	17.4
$75,001–100,000	32	20.7
>$100,0000	60	38.7
Marital status (*n* = 155)	
Married/partnered	122	78.7
Divorced/separated	25	16.1
Single/never married	4	2.6
Widowed	4	2.6
Mothers' FXS Status	
Do not have the full or premutation	7	4.5
Not tested or I don't know	17	11.0
Premutation	127	81.9
Missing	4	2.6
CGG repeats (*n* = 155)	
55–69	9	5.8
70–79	17	11.0
80–89	13	8.4
90–99	14	9.0
100–150	23	14.8
151+	9	5.8
Missing	70	45.2

There were 322 children represented by the 155 mothers; respondents had an average of 2.1 children (SD = 0.9; range = 1–4), and an average of 1.3 children with FXS (SD = 0.6; range = 1–3). Only nine families reported having at least one child with the PM; these 9 families had between 1 and 3 children with the PM. The mean number of co-occurring conditions among all children in the family was 3.1 (SD = 2.6, range = 0–9), the mean number of co-occurring conditions among families (total among all children) was 6.4 (SD = 3.7, range = 0–21) and mean age was 18.5 years (SD = 6.4, range = 2.4–44.2). Approximately 35% of the children were female.

Compared to those in the larger database that were invited to the survey but did not participate (N = 551), those that completed the survey were more likely to have higher education (*p* < 0.01) and higher income (*p* < 0.001). There were no differences by age, employment status, or marital status by survey completion.

### Measures

Survey items included a module for women to update basic demographic information regarding family income, education level, number of children with and without FXS, and the mother's CGG repeat number. If they did not know the exact CGG repeat number, we asked them to indicate the range (55–69; 70–79; 80–89; 90–99; 100–150; 150+). A little over half (55%) were able to report on their CGG repeat number.

#### Severity of child disability

As a proxy for the severity of the children's disability, we used a count of the number of co-occurring conditions for which each child in the family has been diagnosed and treated, Mothers responded whether each child has been diagnosed and treated for autism, anxiety, hyperactivity, aggression, self-injury, attention disorder, seizures, depression, and/or developmental disability. The total number of co-occurring conditions among children listed in the family were summed regardless of the child's FXS status. Although not a direct measure of child behavior challenges, this variable has been used in previous studies as a stand in for the severity of the children's disability (42–47) and was strongly correlated with measures of child behavior in previous survey studies (0.65, *p* < 0.001).

#### Stress

The Perceived Stress Scale [PSS; (48)] was used as a primary measure of stress. The PSS is a 10-item measure that assesses an individual's perception regarding how unpredictable, uncontrollable and overloaded respondents find their lives to be in the past month. Previous studies show good psychometrics, including correlations with other measures of stress (49).

#### Anxiety and depression

The National Institutes of Health (NIH) Patient-Reported Outcome Measurement Information System (PROMIS) measures of anxiety and depression (50) were used for this study. PROMIS measures have been developed and validated to be psychometrically sound for use in diverse research and clinical settings. The PROMIS short form v1.0-Anxiety 8a measure was used in this study. This version consists of eight items assessing self-reported fear, anxious misery, hyperarousal, and somatic symptoms of anxiety. To measure depressive symptoms, the PROMIS short form v1.0-Depression 8a was used to assess self-reported negative mood, views of self, social cognition, and decreased positive affect and engagement.

#### Physical health symptoms

To assess physical health, a series of 12 items assessing the frequency of physical symptoms experienced in the past month were administered. These items have been previously used to characterize physical health in women with an *FMR1* premutation (16, 19).

#### Trait mindfulness

The Five Factor Mindfulness Questionnaire–Short Form [FFMQ-SF; (51)] is a validated short version of the frequently used, 39-item FFMQ. The FFMQ-SF assesses general mindfulness in everyday life. It measures five constructs of dispositional mindfulness (observing, describing, acting with awareness, nonreactivity, and accepting without judgement). These constructs can be combined into a total mindfulness score. The FFMQ has good psychometric properties for both meditating and nonmeditating samples (52); the internal consistency for FFMQ items was high in this sample (Cronbach's alpha = 0.86). Higher FFMQ scores characterize more mindfulness in everyday life.

#### Mindful parenting

To assess mindfulness in the parenting role, we used the Bangor Mindful Parenting Scale [BMPS; (41)]. The BMPS was based on the FFMQ, with similar underlying constructs (observing, describing, acting with awareness, nonreactivity, and accepting without judgement) measured within the context of parenting. It was found to have good psychometric properties in a study of parents of children with autism (41); the internal consistency for BMP items was also high in this sample (Cronbach's alpha = 0.83). Higher BMP scores represent more mindful parenting.

#### Psychological acceptance

The seven-item Acceptance and Action Questionnaire-II [AAQ-II; (53)] was used. The AAQ-II measures the willingness to experience (i.e., not alter the form, frequency, or sensitivity) unwanted events in the pursuit of one's values and goals. Satisfactory structure, reliability, and validity of this measure have been reported for a community-based sample (53). For the current sample, the internal consistency for items on the AAQ was very high (Cronbach's alpha = 0.94). Lower AAQ-II scores indicate higher psychological acceptance and flexibility.

See Table 2 for means for the sample across all measures.

**Table 2:** Description of measures.

		**Observed data**		**Means of imputed datasets (*n* = 155)**
**Mindfulness predictor**	**Measured by**	***N***	**Mean (SD)**	**Mean (SD)**
General mindfulness	The Five Factor Mindfulness Questionnaire-Short Form (FFMQ) (51)	143	79.70 (12.86)	79.81 (12.50)
Mindfulness in parenting	Bangor Mindful Parenting Scale (BMPS) (41)	143	32.26 (5.93)	32.19 (5.70)
Acceptance/flexibility	Acceptance and Action Questionnaire-II (AAQ) (53)	142	16.92 (9.50)	17.15 (9.40)
**Maternal health outcome**	**Measured by**	***N***	**Mean (SD)**	**Mean (SD)**
Stress	The Perceived Stress Scale (48)	145	16.99 (7.58)	17.08 (7.39)
Anxiety	NIH Patient-Reported Outcome Measurement Information System measure of anxiety (PROMIS)	144	19.68 (7.39)	19.84 (7.31)
Depression	NIH Patient-Reported Outcome Measurement Information System measure of depression (PROMIS)	144	13.86 (6.34)	13.98 (6.24)
Daily health symptoms	A 12-item scale measuring the frequency of physical health symptoms in the past month (16, 19)	144	9.42 (5.36)	9.39 (5.20)

### Analysis

The primary objective of this analysis was to test whether trait mindfulness, mindful parenting, or acceptance/flexibility was significantly associated with improved maternal health outcomes, and whether controlling for cross-sectional measures of various mindfulness measures attenuated the effect between the severity of children's disability (total co-occurring conditions) and maternal outcomes in mothers of children with FXS. Because we are utilizing cross-sectionally collected data, we do not test the mindfulness measures as potential mediators between the severity of children's disability and maternal health outcome, nor can we determine that mindfulness practices precede health outcomes, as we cannot establish the temporal relationships between these domains with the available data. However, a significant relationship between the mindfulness dimension and the maternal health outcome and a moderate reduction in the magnitude of the effect of disability severity may support that the mindfulness dimension has protective effects against the stressors of having a child or children with disabilities.

A series of stepwise ordinary least squares (OLS) regression models were used to test for the association between (1) children's disability severity and maternal stress, anxiety, depression, and physical health (2) children's disability severity and maternal outcomes after separately controlling for the three components of mindfulness.

Statistical analyses were conducted using SAS Enterprise Guide 7.15 (Cary, NC). For 13 of the 14 independent and dependent variables of interest, rates of missing data ranged from 0 to 8%. The rate of missing data for CGG repeat category was much higher, at 45%. Multiple imputation (MI) procedures (25 imputed datasets) were used to generate complete demographic data (rates of missing data: mother's age and income = 1%), maternal mindfulness data (rates of missing data for FFMQ, BMP, and AAQ = 8%), and maternal outcome data (rates of missing data for stress, anxiety, depression, and physical health = 7%).

First, covariates of interest including maternal age, child's age, maternal education, marital status (married vs. not), income, CGG repeat, number of children, and children's disability severity (total co-occurring conditions) were examined in correlation matrices with the four outcomes of interest. Correlations were examined within observed (with missing data) and complete (via imputed) data. Maternal-level variables that were significantly related to the maternal outcome at the *p* = 0.05 level in the imputed dataset were included as covariates in their respective models (correlations using observed and imputed data provided the same inferences). The number of children in the family was always entered as a covariate in subsequent analyses.

To ease interpretability and allow for more direct comparisons across mindfulness measures in the regression models, continuous variables were standardized so that mean = 0 and standard deviation = 1.

In Step 1, each maternal health outcome variable was regressed onto the severity of children's disability while controlling for the covariates of interest from the correlation matrix. In step 2, each maternal health outcome was regressed onto the severity of children's disability, controlling for covariates of interest from the correlation matrix and the respective mindfulness measure: model A controlled for general mindfulness (FFMQ), model B controlled for mindfulness in parenting (BMP), and model C controlled for acceptance and flexibility (AAQ).

Due to the use of MI, estimates in each of the 25 imputed datasets were pooled across models using PROC MI ANALYZE. Mean adjusted R-squared values were calculated to estimate the total variance explained by each model. Employing the Bonferroni correction for multiple tests (12 tests), a *p* < 0.004 (alpha = 0.05/12) for the dimensions of mindfulness was considered statistically significantly different from zero.

To explore the change in magnitude before and after controlling for the mindfulness dimension, a percent difference score was calculated [(β before adjustment–β after adjustment)/β before adjustment x 100].

## Results

### Preliminary analysis

Perceived stress for this sample was high relative to stress in the general population [5.3%; (54)] with 38% reporting elevated stress levels according to the PSS (observed mean = 16.99; SD = 7.58). Anxiety was relatively high in this sample, with nearly half (43%) reporting elevated symptoms based on guidelines from the PROMIS measures. Depression was not as much of a concern, with 14% reporting clinically elevated symptoms based on guidelines from the PROMIS measures. Daily health symptoms were similar to other reports of this measure [(16, 19); observed mean = 9.42, SD = 5.36] with around a quarter (22%) of women reporting more physical symptoms than expected based on the general population of women.

### Testing the hypotheses

None of the maternal demographic variables, including CGG repeat length, were significantly correlated with stress or anxiety. Maternal education was correlated with depression (*r* = −0.20, *p* = 0.01) and daily health symptoms (*r* = −0.18, *p* = 0.02), and marital status was correlated with depression (*r* = −0.21, *p* = 0.009) and were thus retained for their respective regression models. The total number of co-occurring conditions in the family was correlated with anxiety, depression, and daily health symptoms (Table 3).

**Table 3:** Correlations among means of imputed datasets.

	**Observed data**				**Means of imputed datasets**			
	**Stress**	**Anxiety**	**Depression**	**Physical health**	**Stress**	**Anxiety**	**Depression**	**Physical health**
Mother's age	−0.12	−0.09	−0.01	−0.14	−0.10	−0.08	0.01	−0.14
Child's age	−0.14	−0.09	−0.003	−0.05	−0.13	−0.08	0.004	−0.05
Marital Status	−0.15	−0.14	−0.20^*^	−0.02	−0.15	−0.14	−0.21^**^	0.0001
CGG repeat^a^	−0.03	−0.03	0.01	0.04	−0.03	−0.03	0.001	0.04
Mother's education	−0.03	−0.02	−0.21^*^	−0.19^*^	−0.02	−0.002	−0.20^*^	−0.18^*^
Income	−0.07	−0.02	−0.13	−0.08	−0.05	−0.03	−0.15	−0.07
Number of children in family	−0.03	0.01	0.04	0.05	−0.04	−0.01	0.03	0.05
Number of children with FM/PM	−0.11	−0.07	−0.02	0.04	−0.11	−0.07	−0.03	0.04
Total Co-occurring conditions in Family^b^	0.15	0.21^*^	0.21^*^	0.32^***^	0.15	0.22^**^	0.21^**^	0.32^***^

a *CGG repeat variable not imputed, n = 85*.

b *Total number of co-occurring conditions among all children in family*.

* *p < 0.05*,

** *p < 0.01*,

*** *p < 0.001*.

See Table 4 for a summary of the OLS regression results. Each set of predictors were jointly predictive of each maternal outcome. The severity of children's disability significantly predicted each maternal outcome after controlling for total number of children in the family and where necessary, education and/or marital status: maternal stress: β = 0.18, *p* = 0.04, maternal anxiety: β = 0.24, *p* = 0.004, maternal depression: β = 0.18, *p* = 0.04, and daily symptoms: β = 0.32, *p* < 0.001 (Table 4, Step 1: No Mindfulness).

**Table 4:** Stepwise regression results: general mindfulness as protective factor.

	**Step 1: no Mindfulness**	**Step 2, Model A: general mindfulness**	**Step 2, Model B: mindfulness in parenting**	**Step 2, Model C: acceptance/flexibility**
	β **(95% CI)**	β **(95% CI)**	β **(95% CI)**	β **(95% CI)**
**OUTCOME IS STRESS**				
Number of children in family	−0.10 (−0.27, 0.07)	−0.06 (−0.20, 0.08)	−0.12 (−0.27, 0.04)	−0.04 (−0.17, 0.09)
Total co-occurring conditions	0.18 (0.01, 0.35)^*^	0.15 (0.01, 0.29)^*^	0.14 (−0.01, 0.29)	0.03 (−0.10, 0.16)
Dimension of Mindfulness	–	−0.58 (−0.72, −0.44)^***^	–0.45 (−0.60, −0.30)^***^	0.67 (0.55, 0.79)^***^
*R^2^*	0.03	0.36	0.23	0.46
*F (NDF, DDF)*	2.15 (2, 16.091)	26.24 (3, 10543)^***^	14.09 (3, 22848)^***^	40.05 (3, 11998)^***^
**OUTCOME IS ANXIETY**				
Number of children in family	−0.09 (−0.26, 0.08)	−0.05 (−0.19, 0.09)	−0.10 (−0.26, 0.06)	−0.03 (−0.16, 0.10)
Total co-occurring conditions	0.24 (0.08, 0.41)^**^	0.21 (0.08, 0.35)^**^	0.22 (0.06, 0.38)^**^	0.10 (−0.04, 0.23)
Dimension of Mindfulness	–	−0.58 (−0.72, −0.45)^***^	−0.27 (−0.43, −0.12)^***^	0.67 (0.55, 0.79)^***^
*R^2^*	0.05	0.39	0.13	0.49
*F (NDF, DDF)*	3.98 (2, 17001)^*^	29.96 (3, 12616)^***^	6.96 (3, 17304)^***^	44.51 (3, 12083)^***^
**OUTCOME IS DEPRESSION**				
Mother's education	−0.17 (−0.33, −0.02)^*^	−0.16 (−0.29, −0.03)^*^	−0.20 (−0.35, −0.06)^**^	−0.12 (−0.24, −0.01)^*^
Mother's marital status (married)	−0.41 (−0.81, −0.02)^*^	−0.38 (−0.70, −0.06)^*^	−0.29 (−0.67, 0.08)	−0.14 (−0.43, 0.15)
Number of children in family	−0.04 (−0.21, 0.13)	−0.01 (−0.14, 0.14)	−0.07 (−0.23, 0.09)	0.01 (−0.12, 0.13)
Total co-occurring conditions	0.18 (0.01, 0.35)^*^	0.15 (0.01, 0.29)^*^	0.16 (0.004, 0.32)^*^	0.06 (−0.07, 0.18)
Dimension of Mindfulness	–	−0.55 (−0.69, −0.41)^***^	−0.34 (−0.49, −0.19)^***^	0.67 (0.56, 0.80)^***^
*R^2^*	0.10	0.41	0.22	0.53
*F (NDF, DDF)*	4.05 (4, 27619)^**^	18.67 (5, 16340)^***^	7.71 (5, 35938)^***^	31.18 (5, 25909)^***^
**OUTCOME IS DAILY HEALTH**				
Mother's education	−0.16 (−0.31, −0.01)^*^	−0.15 (−0.30, −0.01)^*^	−0.17 (−0.32, −0.01)^*^	−0.12 (−0.26, 0.01)
Number of children in family	−0.08 (−0.25, 0.08)	−0.06 (−0.22, 0.10)	−0.09 (−0.25, 0.08)	−0.04 (−0.19, 0.11)
Total co-occurring conditions	0.32 (0.16, 0.48)^***^	0.30 (0.15, 0.46)^***^	0.31 (0.15, 0.47)^***^	0.23 (0.08, 0.38)^**^
Dimension of Mindfulness	–	−0.31 (−0.47, −0.14)^***^	−0.09 (−0.25, 0.07)	0.42 (0.28, 0.57)^***^
*R^2^*	0.12	0.22	0.13	0.29
*F (NDF, DDF)*	6.73 (3, 40764)^***^	9.63 (4, 12001)^***^	5.25 (4, 19795)^***^	14.82 (4, 32974)^***^

* *p < 0.05*,

** *p < 0.01*,

*** *p < 0.001*.

#### General mindfulness (FFMQ)

General mindfulness, as measured by the FFMQ, was a significant predictor of all four maternal outcomes (Table 4, Step2: Model A), such that increased general mindfulness was related to lower scores on measures of stress, (β = −0.58, *p* < 0.001), anxiety (β = −0.58, *p* < 0.001), depression (β = −0.55, *p* < 0.001), and daily symptoms (β = −0.31, *p* < 0.001). The severity of children's disability continued to be significantly related to stress (β = 0.15, *p* = 0.04), anxiety (β = 0.21, *p* = 0.002), depression (β = 0.15, *p* = 0.03), and daily health symptoms (β = 0.30, *p* < 0.001) after including general mindfulness in the model. The effect of disability on each maternal outcome decreased slightly when general mindfulness was added to the model: effects reduced by 16.67% for stress, 12.50% for anxiety, 16.67% for depression, and 6.25% for daily health symptoms.

The total variation explained by each model of maternal health outcome increased moderately when mindfulness in parenting was added to the model, with the largest increase shown for the outcome of maternal anxiety: adjusted R^2^ = 0.05 in the unadjusted model and adjusted R^2^ = 0.39 in the model adjusted by general mindfulness.

#### Mindfulness in parenting (BMP)

Mindfulness in parenting was a significant predictor of three maternal outcomes, such that higher mindful parenting was related to lower scores on measures of stress (β = −0.45, *p* < 0.001), anxiety (β = −0.27, *p* = 0.001), and depression (β = −0.34, *p* < 0.001). The severity of children's disability continued to be related to anxiety (β = 0.22, *p* = 0.007), depression (β = 0.16, *p* = 0.05), and daily health symptoms (β = 0.31, *p* < 0.001) after including mindfulness in parenting in the model; the effect of disability on stress was no longer statistically significant at the *p* = 0.05 level (β = 0.14, *p* = 0.07). The effect of disability on each maternal outcome decreased when mindfulness in parenting was added to the model: effects reduced by 22.22% for stress, 8.33% for anxiety, 11.11% for depression, and 3.13% for daily health symptoms.

The total variation explained by each model of maternal health outcome increased slightly when mindfulness in parenting was added to the model, with the largest increase shown for the outcome of maternal stress: adjusted R^2^ = 0.03 in the unadjusted model and adjusted R^2^ = 0.23 in the model adjusted by mindfulness in parenting.

#### Acceptance and flexibility (AAQ)

Acceptance and flexibility significantly predicted all maternal outcomes, such that better acceptance and flexibility was associated with lower stress (β = 0.67, *p* < 0.001), anxiety (β = 0.67, *p* < 0.001), depression (β = 0.67, *p* < 0.001), and daily symptoms (β = 0.42, *p* < 0.001).

The effect of children's disability severity on stress (β = 0.03, *p* = 0.63), anxiety (β = 0.10, *p* = 0.14), and depression (β = 0.06, *p* = 0.38) were no longer statistically significant at the *p* = 0.05 level when controlling for acceptance/flexibility. The severity of children's disability was still significantly associated with the mother's daily health symptoms (β = 0.23, *p* = 0.002) after controlling for acceptance/flexibility. The effect of disability on each maternal outcome decreased moderately to substantially when acceptance/flexibility was added to the model: 83.33% for stress, 58.33% for anxiety, 66.67% for depression, and 28.13% for daily health symptoms.

The total variation explained by each model of maternal health outcome often increased substantially when acceptance/flexibility was added to the model, with the largest increase shown for the outcome of maternal stress: R^2^ = 0.03 in the unadjusted model and R^2^ = 0.46 in the model adjusted by acceptance/flexibility. Further, the total variation explained by the model that included acceptance or flexibility was larger for the outcomes of maternal stress (R^2^ = 0.46), anxiety (R^2^ = 0.49), and depression (R^2^ = 0.53) and daily health (R^2^ = 0.29) than models of the same outcomes that included mindfulness in parenting (R^2^ for stress = 0.23, for anxiety = 0.13, for depression = 0.22, for daily health = 0.13) or general mindfulness (R^2^ for stress = 0.36, for anxiety = 0.39, for depression = 0.41, for daily health = 0.22).

## Discussion

The primary goal of this study was to explore potential psychological processes that may serve as protective factors for women experiencing the dual vulnerability of having an *FMR1* premutation and parenting one or more children with FXS. A growing body of research shows that individuals with a premutation may have an increased biological risk for stress-related illnesses. Thus, understanding mechanisms that may be protective is of paramount importance and are critical for designing effective intervention strategies.

Our findings are consistent with previous research on parents of children with ID or autism (38–41). Specifically, acceptance was related to improved maternal stress, anxiety, and depression, and controlling for acceptance/flexibility lead to an attenuated relationship between child severity of disability and maternal health symptoms. Acceptance as a psychological construct reflects one's ability to take what is offered from life without trying to avoid experiences (55). Theoretically, greater levels of acceptance within the context of parenting a child with a disability suggests an ability to manage the challenges of child-rearing while remaining open and flexible to the experience of parenting. Improving the ability to cope and problem-solve while simultaneously accepting difficult thoughts and feelings is a key feature of many acceptance-based treatments (22, 56). In addition, preliminary evidence suggests that an intervention approach focusing on increasing acceptance can make a difference in the quality of life for both caregivers and the individual with special needs (57–59). These studies involved short term (1–2 day) workshops that provided didactic teaching, group discussions, and practical and interactive exercises based on the core principles of Acceptance and Commitment Therapy. While the sample sizes were small for these trials, the positive results suggest a strong potential for improving outcomes for parents of children with FXS through the inclusion of practices that increase psychological acceptance.

It is important to note that we used a measure of general acceptance, as opposed to one specifically for parents of children with an intellectual disability, which has been used in previous studies [e.g., (41)]. However, our results were similar, suggesting that those who are higher in overall psychological acceptance likely apply that to the experience of parenting a child with an intellectual disability. However, a deeper exploration, perhaps through a mixed qualitative-quantitative approach into how mothers of children with FXS describe their levels of general acceptance and acceptance of their child's disability is warranted.

Also, as seen in previous findings, mindfulness and mindful parenting were both significantly related to maternal stress, depression, and anxiety. Trait mindfulness and mindful parenting were highly correlated, suggesting, as concluded by Jones et al. (41), that parents who are generally mindful are also mindful in the parenting context. However, while trait mindfulness was related to physical health, mindful parenting was not, suggesting a potentially different pathway by which general mindfulness may impact health.

It has been suggested that improvements in mindfulness and acceptance impact psychological and physical outcomes not directly, but rather indirectly through changing how individuals cope with stressors and appraise threats in their environment (60–62). For example, Weinstein et al. (63) found the individuals high in mindfulness were more likely to use healthier coping strategies when faced with stressors and were more likely to see potentially stressful events in their lives as benign. Consistent with the differential susceptibility hypothesis (64), women with a premutation—who are at increased genetic risk for stress vulnerability—may be especially responsive to interventions that promote coping strategies to manage stress. Future research on coping strategies used by mothers of children with FXS and how the constructs of mindfulness and acceptance are related to coping will be important to understand who is most likely to benefit from mindfulness-based intervention techniques.

### Limitations and future directions

The sample was non-representative, with primarily white, well-educated women responding; therefore, the generalizability of the findings is limited. We were also not able to get confirmation of genetic status and do not have independent reports from medical providers of the endorsed co-occurring conditions. Our measure of co-occurring conditions may be a conservative measure, as children may also have additional conditions not included. Data are concurrent, so causality and the direction of effects cannot be inferred. It may be that those with higher levels of mindfulness and acceptance have greater emotional and physical health or that those who are healthier are able to practice more mindfulness and acceptance. Finally, because all measures are completed by the same participant, there is a possibility that there is an inflation of effects due to response bias.

Despite these limitations, this study provides an important peek into the psychological processes that may prove to be protective for women with an *FMR1* premutation. Future directions include a deeper exploration with a larger, more diverse sample on these mechanisms to better understand how increased mindfulness and acceptance impact health outcomes and a longitudinal study to explore the direction of effect and impact on both child and parent outcomes. These results can inform the development of interventions that could be applied to individuals with a premutation in the hopes of improving long-term health outcomes for *FMR1*-premutation carriers.

## Ethics statement

This study was carried out in accordance with the recommendations of the RTI Institutional Review Board with electronic informed consent from all subjects. All subjects gave written electronic informed consent in accordance with the Declaration of Helsinki. The protocol was approved by the RTI Institutional review board.

## Author contributions

AWh conceptualized the study, drafted most of the paper, and approved the final version. SM contributed to the conceptualization to the study and reviewed and approved the final version. AWy provided all the statistical analysis, wrote the data analysis section, and approved the final version. AE provided input on the design of the survey, supported the recruitment of participants, and reviewed and approved the final version of the manuscript.

### Conflict of interest statement

The authors declare that the research was conducted in the absence of any commercial or financial relationships that could be construed as a potential conflict of interest.

## References

[1] Miodrag N Hodapp RM. Chronic stress and health among parents of children with intellectual and developmental disabilities. Curr Opin Psychiatr. (2010) 23:407–11. 10.1097/YCO.0b013e32833a8796 20592593

[2] Bailey DB Jr Raspa M Olmsted M Holiday DB. Co-occurring conditions associated with FMR1 gene variations: findings from a national parent survey. Am J Med Genet Part A (2008) 146A:2060–9. 10.1002/ajmg.a.32439 18570292

[3] Bailey DB Raspa M Bishop E Mitra D Martin S Wheeler A. Health and economic consequences of fragile X syndrome for caregivers. J Dev Behav Pediatr. (2012) 33:705–12. 10.1097/DBP.0b013e318272dcbc 23117595

[4] Wheeler A Raspa M Bishop E Bailey DB. Aggression in fragile X syndrome. J Intellect Disabil Res. (2015) 60:113–25. 10.1111/jir.12238 26628097

[5] Sullivan SD Welt C Sherman S. FMR1 and the continuum of primary ovarian insufficiency. Sem Reprod Med. (2011) 29:299–307. 10.1055/s-0031-1280915 21969264

[6] Tassone F Hagerman RJ Taylor AK Mills JB Harris SW Gane LW. Clinical involvement and protein expression in individuals with the FMR1 premutation. Am J Med Genet. (2000) 91:144–52. 10.1002/(sici)1096-8628(20000313)91:2<144::aid-ajmg14>3.0.co;2-v 10748416

[7] Wheeler AC Bailey DB Jr Berry-Kravis E Greenberg J Losh M Mailick M. Associated features in females with an FMR1 premutation. J Neurodev Disorders (2014) 6:30. 10.1186/1866-1955-6-30 25097672 PMC4121434

[8] Seltzer MM Barker ET Greenberg JS Hong J Coe C Almeida D. Differential sensitivity to life stress in FMR1 premutation carrier mothers of children with fragile X syndrome. Health Psychol. (2012) 31:612–22. 10.1037/a0026528 22149120 PMC3434309

[9] Allen EG Sullivan AK Marcus M Small C Dominguez C Epstein MP. Examination of reproductive aging milestones among women who carry the FMR1 premutation. Hum Reprod. (2007) 22:2142–52. 10.1093/humrep/dem148 17588953

[10] Ennis S Ward D Murray A. Nonlinear association between CGG repeat number and age of menopause in FMR1 premutation carriers. Eur J Hum Genet. (2006) 14:253–5. 10.1038/sj.ejhg.5201510 16251893 PMC3725422

[11] Sullivan AK Marcus M Epstein MP Allen EG Anido AE Paquin JJ. Association of FMR1 repeat size with ovarian dysfunction. Hum Reprod. (2005) 20:402–12. 10.1093/humrep/deh635 15608041

[12] Tejada MI Garcia-Alegria E Bilbao A Martínez-Bouzas C Beristain E Poch M. Analysis of the molecular parameters that could predict the risk of manifesting premature ovarian failure in female premutation carriers of fragile X syndrome. Menopause (2008) 15:945–9. 10.1097/gme.0b013e3181647762 18427356

[13] Hunter JE Rohr JK Sherman SL. Co-occurring diagnoses among FMR1 premutation allele carriers. Clin Genet. (2010) 77:374–81. 10.1111/j.1399-0004.2009.01317.x 20059484 PMC3696492

[14] Loesch DZ Bui MQ Hammersley E Schneider A Storey E Stimpson P. Psychological status in female carriers of premutation FMR1 allele showing a complex relationship with the size of CGG expansion. Clin Genet. (2015) 87:173–8. 10.1111/cge.12347 24428240 PMC4115039

[15] Coffey SM Cook K Tartaglia N Tassone F Nguyen DV Pan R. Expanded clinical phenotype of women with the FMR1 premutation. Am J Med Genet Part A (2008) 146A:1009–16. 10.1002/ajmg.a.32060 18348275 PMC2888464

[16] Smith LE Seltzer MM Greenberg JS. Daily health symptoms of mothers of adolescents and adults with fragile x syndrome and mothers of adolescents and adults with autism spectrum disorder. J Autism Dev Disorders (2012) 42:1836–46. 10.1007/s10803-011-1422-7 22167342 PMC3426638

[17] Brouwer JR Severijnen E de Jong FH Hessl D Hagerman RJ Oostra BA. Altered hypothalamus-pituitary-adrenal gland axis regulation in the expanded CGG-repeat mouse model for fragile X-associated tremor/ataxia syndrome. Psycho Neuroendocrinol. (2008) 33:863–73. 10.1016/j.psyneuen.2008.03.011 18472227 PMC4408208

[18] Roberts JE Bailey DB Mankowski J Ford A Sideris J Weisenfeld LA. Mood and anxiety disorders in females with the FMR1 premutation. Am J Med Genet Part B (2009) 150B:130–9. 10.1002/ajmg.b.30786 18553360

[19] Wheeler AC Raspa M Green A Bishop E Bann C Edwards A. Health and reproductive experiences of women with an FMR1 premutation with and without fragile X premature ovarian insufficiency. Front Genet. (2014) 5:300. 10.3389/fgene.2014.00300 25250044 PMC4157548

[20] Hartley SL Seltzer MM Hong J Greenberg JS Smith L Almeida D. Cortisol response to behavior problems in FMR1 premutation mothers of adolescents and adults with fragile X syndrome: a diathesis-stress model. Int J Behav Dev. (2012) 36:53–61. 10.1177/0165025411406857 22798702 PMC3396207

[21] Polussa J Schneider A Hagerman R. Molecular advances leading to treatment implications for Fragile X premutation carriers. Brain Disorders Ther. (2014) 3:1000119. 10.4172/2168-975X.1000119 25436181 PMC4245015

[22] Hayes SC Strosahl KD Wilson KG Acceptance and Commitment Therapy: An Experiential Approach to Behavior Change. New York, NY: Guilford Press (1999).

[23] Keng SL Smoski MJ Robins CJ. Effects of mindfulness on psychological health: a review of empirical studies. Clin Psychol Rev. (2011) 31:1041–56. 10.1016/j.cpr.2011.04.006 21802619 PMC3679190

[24] Chiesa A Serretti A. Mindfulness-based stress reduction for stress management in healthy people: a review and meta-analysis. J Altern Complement Med. (2009) 15:593–600. 10.1089/acm.2008.0495 19432513

[25] Benn R Akiva T Arel S Roeser RW. Mindfulness training effects for parents and educators of children with special needs. Dev Psychol. (2012) 48:1476. 10.1037/a0027537 22409766

[26] Cachia RL Anderson A Moore DW Mindfulness, stress and well-being in parents of children with autism spectrum disorder: a systematic review. J Child Family Stud. (2016) 25:1–4. 10.1007/s10826-015-0193-8

[27] Noone SJ Hastings RP. Building psychological resilience in support staff caring for people with intellectual disabilities: pilot evaluation of an acceptance-based intervention. J Intellect Disabil. (2009) 13:43–53. 10.1177/1744629509103519 19332508

[28] Noone SJ Hastings RP Using acceptance and mindfulness-based workshops with support staff caring for adults with intellectual disabilities. Mindfulness (2010) 1:67–73. 10.1007/s12671-010-0007-4

[29] Singh NN Lancioni GE Winton AS Fisher BC Wahler RG Mcaleavey K Mindful parenting decreases aggression, noncompliance, and self-injury in children with autism. J Emotional Behav Disorders (2006) 14:169–77. 10.1177/10634266060140030401

[30] Singh NN Lancioni GE Winton AS Adkins AD Wahler RG Sabaawi M. Individuals with mental illness can control their aggressive behavior through mindfulness training. Behav Mod. (2007) 31:313–28. 10.1177/0145445506293585 17438345

[31] Hofmann SG Sawyer AT Witt AA Oh D. The effect of mindfulness-based therapy on anxiety and depression: a meta-analytic review. J Consult Clin Psychol. (2010) 78:169. 10.1037/a0018555 20350028 PMC2848393

[32] Garland EL Gaylord SA Boettiger CA Howard MO. Mindfulness training modifies cognitive, affective, and physiological mechanisms implicated in alcohol dependence: results of a randomized controlled pilot trial. J Psychoact Drugs (2010) 42:177–92. 10.1080/02791072.2010.10400690 20648913 PMC2921532

[33] Astin JA. Stress reduction through mindfulness meditation. Psychother Psychosomat. (1997) 66:97–106. 10.1159/000289116 9097338

[34] Grossman P Niemann L Schmidt S Walach H. Mindfulness-based stress reduction and health benefits: a meta-analysis. J Psychosomat Res. (2004) 57:35–43. 10.1016/S0022-3999(03)00573-7 15256293

[35] Gross CR Kreitzer MJ Reilly-Spong M Wall M Winbush NY Patterson R. Mindfulness-based stress reduction versus pharmacotherapy for chronic primary insomnia: a randomized controlled clinical trial. Explore (2011) 7:76–87. 10.1016/j.explore.2010.12.003 21397868 PMC3077056

[36] Blackledge JT Hayes SC Using acceptance and commitment training in the support of parents of children diagnosed with autism. Child Family Behav Ther. (2006) 28:1–18. 10.1300/j019v28n01_01

[37] Singh NN Lancioni GE Winton AS Karazsia BT Myers RE Latham LL Mindfulness-based positive behavior support (MBPBS) for mothers of adolescents with autism spectrum disorder: effects on adolescents' behavior and parental stress. Mindfulness (2014) 5:646–57. 10.1007/s12671-014-0321-3

[38] MacDonald EE Hastings RP Fitzsimons E Psychological acceptance mediates the impact of the behaviour problems of children with intellectual disability on fathers' psychological adjustment. J Appl Res Intellect Disabil. (2010) 23:27–37. 10.1111/j.1468-3148.2009.00546.x

[39] Lloyd T Hastings RP. Psychological variables as correlates of adjustment in mothers of children with intellectual disabilities: cross-sectional and longitudinal relationships. J Intellect Disabil Res. (2008) 52:37–48. 10.1111/j.1365-2788.2007.00974.x 18173571

[40] Weiss JA Cappadocia MC MacMullin JA Viecili M Lunsky Y. The impact of child problem behaviors of children with ASD on parent mental health: the mediating role of acceptance and empowerment. Autism (2012) 16:261–74. 10.1177/1362361311422708 22297202

[41] Jones L Hastings RP Totsika V Keane L Rhule N. Child behavior problems and parental well-being in families of children with autism: the mediating role of mindfulness and acceptance. Am J Intellect Dev Disabil. (2014) 119:171–85. 10.1352/1944-7558-119.2.171 24679352

[42] Hartley SL Seltzer MM Raspa M Olmstead M Bishop E Bailey DB. Exploring the adult life of men and women with fragile X syndrome: results from a national survey. Am J Intellect Dev Disabil. (2011) 116:16–35. 10.1352/1944-7558-116.1.16 21291308 PMC3238098

[43] Raspa M Bailey DB Bishop E Holiday D Olmsted M. Obesity, food selectivity, and physical activity in individuals With fragile X syndrome. Am J Intellect Dev Disabil. (2010) 115:482–95. 10.1352/1944-7558-115.6.482 20946001

[44] Raspa M Bailey DB Bann C Bishop E. Modeling family adaptation to fragile X syndrome. Am J Intellect Dev Disabil. (2014) 119:33–48. 10.1352/1944-7558-119.1.33 24450320

[45] Raspa M Edwards A Wheeler AC Bishop E Bailey DB. Family communication and cascade testing for fragile X syndrome. J Genet Counseling (2016) 25:1075–84. 10.1007/s10897-016-9940-2 26961978

[46] Kidd SA Raspa M Clark R Usrey-Roos H Wheeler AC Liu JA. Attendance at fragile X specialty clinics: facilitators and barriers. Am J Intellect Dev Disabil. (2017) 122:457–75. 10.1352/1944-7558-122.6.457 29115871

[47] Nash R Riley C Gilbertson K A description of the educational setting among individuals with fragile X syndrome. Am J Intellect Dev Disabil. (in press). 10.1352/1944-7558-124.1.57 PMC6442477 30715925

[48] Cohen S Janicki-Deverts D Who's stressed? Distributions of psychological stress in the United States in probability samples from 1983, 2006, and 2009. J Appl Soc Psychol. (2012) 42:1320–34. 10.1111/j.1559-1816.2012.00900.x

[49] Cohen S Kamarck T Mermelstein R. Perceived stress scale. In: Cohen S Kessler RC Gordon LU, editors. Measuring Stress: A Guide for Health and Social Scientists, Oxford: Oxford University Press (1994). p. 235–83.

[50] Pilkonis PA Choi SW Reise SP Stover AM Riley WT Cella D. Item banks for measuring emotional distress from the Patient-Reported Outcomes Measurement Information System (PROMIS^®;^): depression, anxiety, and anger. Assessment (2011) 18:263–83. 10.1177/1073191111411667 21697139 PMC3153635

[51] Bohlmeijer E ten Klooster PM Fledderus M Veehof M Baer R. Psychometric properties of the five facet mindfulness questionnaire in depressed adults and development of a short form. Assessment (2011) 18:308–20. 10.1177/1073191111408231 21586480

[52] Baer RA Smith GT Lykins E Button D Krietemeyer J Sauer S. Construct validity of the five facet mindfulness questionnaire in meditating and nonmeditating samples. Assessment (2008) 15:329–42. 10.1177/1073191107313003 18310597

[53] Bond FW Hayes SC Baer RA Carpenter KM Guenole N Orcutt HK. Preliminary psychometric properties of the Acceptance and Action Questionnaire-II: a revised measure of psychological inflexibility and experiential avoidance. Behav Ther. (2011) 42:676–88. 10.1016/j.beth.2011.03.007 22035996

[54] American Psychological Association Stress in America: The Impact of Discrimination. Washington, DC: Stress in America™ Survey (2016).

[55] Hayes SC Luoma JB Bond FW Masuda A Lillis J. (2006). Acceptance and commitment therapy: model, processes and outcomes. Behav Res Ther. 44:1–25. 10.1016/j.brat.2005.06.006 16300724

[56] Prevedini AB Presti G Rabitti E Miselli G Moderato P. Acceptance and commitment therapy (ACT): the foundation of the therapeutic model and an overview of its contribution to the treatment of patients with chronic physical diseases. G Ital Med Lav Ergon. (2011) 33(Suppl. 1A):A53–63. Available Online at: http://gimle.fsm.it 21488484

[57] Reid C Gill F Gore N Brady S. New ways of seeing and being: evaluating an acceptance and mindfulness group for parents of young people with intellectual disabilities who display challenging behaviour. J Intellect Disabil. (2016) 20:5–17. 10.1177/1744629515584868 25999396

[58] McConachie DA McKenzie K Morris PG Walley RM. Acceptance and mindfulness-based stress management for support staff caring for individuals with intellectual disabilities. Res Dev Disabil. (2014) 35:1216–27. 10.1016/j.ridd.2014.03.005 24685937

[59] Martin S Wolters PL Toledo-Tamula MA Schmitt SN Baldwin A Starosta A. Acceptance and commitment therapy in youth with neurofibromatosis type 1 (NF1) and chronic pain and their parents: a pilot study of feasibility and preliminary efficacy. Am J Med Genet Part A (2016) 170:1462–70. 10.1002/ajmg.a.37623 27021207 PMC6675568

[60] Baer RA Smith GT Hopkins J Krietemeyer J Toney L. Using self-report assessment methods to explore facets of mindfulness. Assessment (2006) 13:27–45. 10.1177/1073191105283504 16443717

[61] Gross JJ Thompson R Emotion regulation: conceptual foundations. In: Gross JJ, editor. Handbook of Emotion Regulation. New York: Guilford (2013). p. 3–24.

[62] Shapiro SL Brown KW Biegel GM Teaching self-care to caregivers: effects of mindfulness-based stress reduction on the mental health of therapists in training. Training Edu Professional Psychol. (2007) 1:105 10.1037/1931-3918.1.2.105

[63] Weinstein N Brown KW Ryan RM A multi-method examination of the effects of mindfulness on stress attribution, coping, and emotional well-being. J Res Personality (2009) 43:374–85. 10.1016/j.jrp.2008.12.008

[64] Belsky J Pluess M. Beyond diathesis stress: differential susceptibility to environmental influences. Psychol Bull. (2009) 135:885–908. 10.1037/a0017376 19883141

